# Navigating the Landscape of Tumor Extracellular Vesicle Heterogeneity

**DOI:** 10.3390/ijms20061349

**Published:** 2019-03-18

**Authors:** Sabrina Roy, Hsing-Ying Lin, Chung-Yu Chou, Chen-Han Huang, Julia Small, Noah Sadik, Caroline M. Ayinon, Elizabeth Lansbury, Lilian Cruz, Anudeep Yekula, Pamela S. Jones, Leonora Balaj, Bob S. Carter

**Affiliations:** 1 Department of Neurosurgery, Massachusetts General Hospital and Harvard Medical School, Boston, MA 02114, USA; sabrinaroy30@outlook.com (S.R.); jsmall6@mgh.harvard.edu (J.S); noah.sadik@columbia.edu (N.S.); cayinon@mgh.harvard.edu (C.M.A.); elansbury@mgh.harvard.edu (E.L.); ayekula@mgh.harvard.edu (A.Y.); psjones@partners.org (P.S.J.); 2 Center for Systems Biology, Massachusetts General Hospital and Harvard Medical School, Boston, MA 02114, USA; HLIN17@mgh.harvard.edu (H.-Y.L.); 106827015@cc.ncu.edu.tw (C.-Y.C.); 3 Department of Radiology, Massachusetts General Hospital and Harvard Medical School, Boston, MA 02114, USA; 4 Department of Biomedical Sciences and Engineering, National Central University, Taoyuan City 32001, Taiwan; chhuang@ncu.edu.tw; 5 Department of Biomedical Engineering, Columbia University, New York City, NY 10027, USA; 6 Department of Neurology, Massachusetts General Hospital and Harvard Medical School, Boston, MA 02114, USA; lcruz5@mgh.harvard.edu

**Keywords:** extracellular vesicles, heterogeneity, single-cell analysis

## Abstract

The last decade has seen a rapid expansion of interest in extracellular vesicles (EVs) released by cells and proposed to mediate intercellular communication in physiological and pathological conditions. Considering that the genetic content of EVs reflects that of their respective parent cell, many researchers have proposed EVs as a source of biomarkers in various diseases. So far, the question of heterogeneity in given EV samples is rarely addressed at the experimental level. Because of their relatively small size, EVs are difficult to reliably isolate and detect within a given sample. Consequently, standardized protocols that have been optimized for accurate characterization of EVs are lacking despite recent advancements in the field. Continuous improvements in pre-analytical parameters permit more efficient assessment of EVs, however, methods to more objectively distinguish EVs from background, and to interpret multiple single-EV parameters are lacking. Here, we review EV heterogeneity according to their origin, mode of release, membrane composition, organelle and biochemical content, and other factors. In doing so, we also provide an overview of currently available and potentially applicable methods for single EV analysis. Finally, we examine the latest findings from experiments that have analyzed the issue at the single EV level and discuss potential implications.

## 1. Introduction

Extracellular vesicles (EVs) encompass a population of endogenous nano-sized cell-derived membranous vesicles released by eukaryotic and prokaryotic cells [1,2]. Ranging from 30 to 2000 nm in diameter, EVs are released into biofluids by organ-specific cell populations and contain genetic materials reflective of their parent cells [3,4]. EVs are involved in a variety of physiological functions, including immune regulation, stem cell regulation, tissue morphogenesis, and gamete function, but also in pathological states such as cancer progression and neurodegeneration [5,6]. Recently, EVs have been proposed to mediate intercellular communication in pathological and physiological conditions via transfer of their biological content consisting of proteins, lipids, and nucleic acids, between cells [3,7,8,9]. Due to their recent emergence as biomarkers for disease and their construction as targeted therapeutics, interest in the biological roles and clinical applications of EVs is growing rapidly [10,11].

**Figure 1:**
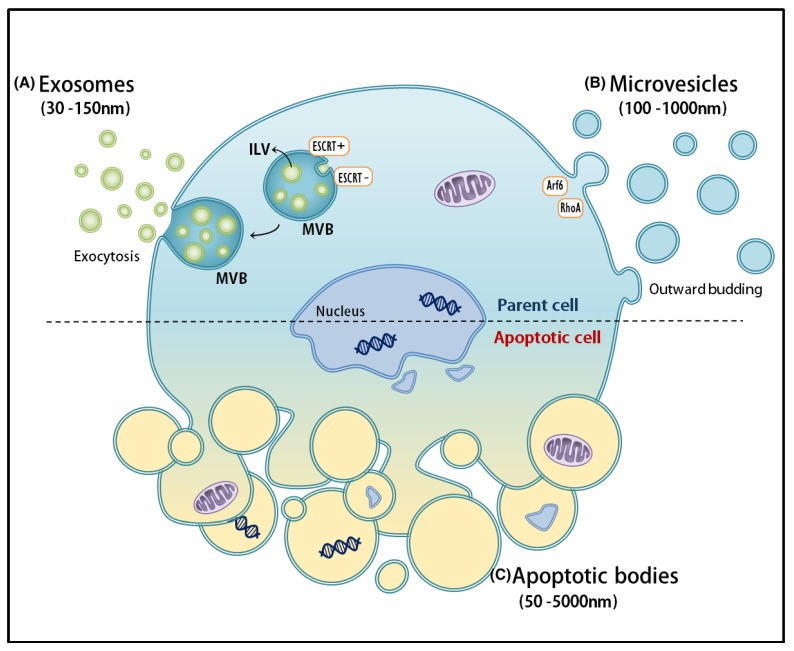
Overview of EV populations. (**A**) Exosomes range in 30–150 nm in diameter and are formed within multivesicular bodies (MVBs), until eventually being released by cells upon fusion with the plasma membrane; (**B**) Microvesicles are released via direct outward budding of the plasma membrane and range from 50–1000 nm in diameter. Important in MV formation and shedding, the protein ARF6 is a key player in the selective incorporation of molecular cargo into MVs. RhoA, a member of the small GTPases family, has been recently identified as a regulator of MV release; (**C**) Apoptotic bodies, formed during cytoskeletal rearrangement, are released through outward blebbing and decomposition of the cell membrane of dying cells, with a large size range of 500–2000 nm in diameter.

Theoretically, EVs can be speciated by their concentrations in biofluid, their cargo, and their functional effects [3]. EVs can also be described in terms of differences in density—in fact, density gradients are widely used during EV isolation to study EV subpopulations and reports indicate that resulting subtype preparations vary in protein, lipid, and RNA content, as well as in biological functions [12,13]. However, differentiation of EV subpopulations at the experimental level remains one of the major challenges within the field [3,14,15]. With regard to their biogenesis pathways, EVs can be categorized into three main classes: exosomes, microvesicles (MVs), and apoptotic bodies (released during apoptosis) (Figure 1). The discovery that perfectly healthy cells also shed vesicles from their plasma membrane has only been widely characterized [16].

Currently, the mainstay of the field includes classification of EVs based on their mode of release or size [15,16]. Exosomes are derived from the endolysosomal pathways and formed within multivesicular bodies (MVBs), until eventually being released by cells upon fusion of MVBs with the plasma membrane [17]. Compared to other classes, exosomes seem to represent a more homogeneous population of EVs, ranging in size from 30 to 150 nm in diameter. Commonly used protein markers for exosomes, such as ALG-2-interacting protein X (ALIX) and tumor susceptibility gene 101 protein (TSG101), are involved in sorting cargo into exosomes and associated with the endosomal sorting complex required for transport (ESCRT) [18].

Despite their seemingly uniform characteristics and the assumption that they represent a relatively consistent population, exosomes mediate a wide array of effects on target cells [4,10]. This either indicates that (1) individual exosomes are highly multifunctional, or that (2) they are characterized by heterogeneity upon their release—supporting the notion that subpopulations of exosomes display distinct compositions and/or functions [3,17,19]. In fact, numerous studies on MVB sorting mechanisms, content, and composition have suggested the potential heterogeneous nature of exosomes [17,19]. For example, since MVBs can form exosomes by relying on ESCRT-dependent and independent pathways, it is suspected that alterations to these pathways could in turn affect MVB dynamics and the subsequent release of exosome subpopulations [20]. MVs, on the other hand, are released via direct outward budding of the plasma membrane and range from 50 to 1000 nm in diameter size. Important in MV formation and shedding, the protein ARF6 is a key player in the selective incorporation of molecular cargo into MVs [21,22]. RhoA, a member of the small GTPases family, has been recently identified as a regulator of MV release [23]. Finally, apoptotic bodies are formed during cytoskeletal rearrangement and released through outward blebbing and decomposition of the cell membrane of apoptotic cells, with a vast size range of 500–2000 nm in diameter [3]. However, many report that the formation of vesicles in the same size range as exosomes can be observed upon analyzing apoptotic cells [14,16]. Although their content is generally thought of as randomly packaged, there is evidence of RNA and DNA sorting into separate distinct apoptotic cell subpopulations [24]. Here we stress that most studies have not clearly defined the origin of EVs [24,25]. Throughout this review, we will use the widely-adopted generic term “extracellular vesicles” (EVs) to refer to an exosome-enriched pool of vesicles, unless stated otherwise. 

It is important to note that there does exist some controversy regarding the nomenclature and size cohorts of the different types of vesicles. Nevertheless, the International Society for Extracellular Vesicles (ISEV) has established a set of criteria for the study of EVs, known as the Minimal Informations for Studies on EVs (MISEV) guidelines [26]. However, considering no real standards have been set to classify vesicle subtypes, researchers must be weary when using size alone in defining vesicle types due to the inherent heterogeneity of all populations [15]. Furthermore, while the clinical interest and relevance of EVs is growing, their isolation and detection remains a challenge [26,27]. In the future, the various biogenesis mechanisms, means of isolation, and EV content may turn out be far more relevant for establishing standard EV characterization criteria and therefore used as the basis for their primary distinction [25]. In this review, we will discuss the question of heterogeneity in given EV samples by addressing current experimental limitations that need to be resolved, as well as highlight the latest findings from experiments looking at single EVs. Methods currently being used for single EV analysis are also outlined. 

## 2. Biogenesis of EVs: Sorting and Heterogeneous Molecular Signatures 

Intraluminal vesicles (ILVs) of MVBs are either sorted for cargo degradation into lysosomes, or secreted as exosomes into the extracellular space. The issue of EV heterogeneity is further complicated by the discovery that different pathways controlling ILV formation in the endosomal compartment have been identified [14]. In fact, ILVs can be formed in two distinct methods: (1) ESCRT-dependent or (2) ESCRT-independent. Following this discovery, speculation rose regarding the potential role of MVB biogenesis-associated machinery in exosome formation. The function of ESCRT components in secretory MVB formation, on the other hand, appears to be far more complex than originally supposed.

When the limiting membrane of the late endosome buds into the lumen, ILVs are formed; within them, a subset of transmembrane proteins and lipids are arranged. When ILVs fuse with lysosomes/vacuoles, both the protein and lipid composition of ILVs are degraded due to exposure to the hydrolytic lumen [28]. In sum, the MVB pathway represents a major transmembrane protein and lipid turnover system in eukaryotic cells [29]. 

Unlike those described in other vesicle formation events, a unique mechanism directed away from the cytoplasm is required for ILV formation. Because ILVs originating from MVBs are generated by inward budding of the endosomal limiting membrane, exosomes consequently have the same topology as MVs and cells, with their exoplasmic side exposed [5]. The ESCRT protein complexes represent the best contenders in terms of machinery responsible for driving MVB vesicle formation. However, increasing evidence points to the notion that lipids may play a key role in this membrane-deformation process [29]. For the majority of membrane proteins that have been studied, luminal vesicle sorting within MVBs is affected by the addition of the small protein ubiquitin to lysine amino acid residues of target proteins (i.e., ubiquitination). ESCRT groups recognize these ubiquitin tags by binding to ubiquitinated cargoes to ensure their proper sorting into ILVs. ESCRT is composed of four multiprotein sub-complexes: ESCRT-0, -I, and -II identify and bind to ubiquitinated membrane proteins, while ESCRT-III drives the budding of ILVs into the lumen [5]. 

Ubiquitination of cargo proteins is so far the most studied determinant for ESCRT-dependent sorting into the extracellular vesicle pathway [30]. However, the sorting of proteins into exosomes appears to occur independently of cargo ubiquitination, and only a select number of ESCRT components are involved in exosome formation. For example, ubiquitination of MHC class II is required for its sorting into ILVs of MVBs targeted by lysosomes, but not for incorporation into exosomes [5,31]. Another example of a process independent to ubiquitination would be the sorting of the transferrin receptor to exosomes, which relies on the attachment of the ESCRT accessory protein ALIX onto its cytoplasmic domain [5]. In studies looking at oligodendroglial cell lines (which secrete the proteo-lipid protein associated with exosomes), exosome biogenesis and secretion do not require ESCRT function but are dependent on sphingomyelinase, an enzyme responsible for the production of the lipid ceramide [5,31]. Such findings are in line with those from other studies which looked at the presence of high concentrations of ceramide and its derivatives in exosomes. In one particular study, purified exosomes were determined to be enriched in ceramide, and the release of exosomes was reduced after the inhibition of neutral sphingomyelinases. These results establish a pathway in intra-endosomal membrane transport and exosome formation. The existence of ESCRT-independent mechanisms for MVB formation is consistent with the finding that cells simultaneously depleted of the four ESCRT complex subunits are still able to generate CD63-positive MVBs; leading to the conclusion that higher eukaryotes employ the established ESCRT system as understood in yeast, and possibly additional ESCRT-independent pathways to form ILVs [24,29]. These ‘unusual’ pathways appear to be prompted by the presence of certain lipids, such as lysobiphosphatidic acid and ceramides. It is hypothesized that these lipids might organize into specialized endosomal portions that bend inward and ultimately form vesicles simply due to local lipid composition [29].

Collectively, there exists several models which attempt at explaining the way in which endosomal membranes detach from the cytoplasm to ultimately form ILVs. However, these models seem to be based on inconsistent findings [29,32,33]. While studies in yeast have established an essential role for the ESCRT machinery in ILV formation [34,35], mammalian cells have been shown to maintain the ability to form ILVs in the absence of key ESCRT components [36]. Furthermore, exosomes have been shown to form independently of ESCRT function [37]. Budding of exosome vesicles has been shown to be dependent on the conversion of sphingomyelin into ceramide by neutral sphingomyelinase [3]. Based on the literature, data suggests that all observed cases of ILV formation rely principally on lipid-driven mechanisms seems plausible, and that the ESCRTs (in the context of MVB sorting) function in regulating the lipid-based reaction and coupling it to cargo sorting.

EV complexity is further multiplied due to the fact that EVs always contain a set of molecules reflecting their cellular origin [25]. Thus, different cell types release different variants of given EV subtypes [16,25]. Finally, the alteration of environmental parameters regularly results in changes in the EVs’ molecular signatures [14].

## 3. Isolation of EVs

Currently, most EVs isolated from the supernatants of cells grown in media containing EV-depleted (by differential centrifugation) fetal bovine serum. After spinning down EVs by means of ultracentrifugation (UC), they must be separated from non-membranous particles (i.e., vesicles that are not of interest, such as protein aggregates) [16]. Due to their relatively low buoyant density and respective differences in floatation velocity, EVs can be efficiently separated based on size and subsequently categorized by different sized classes. While it is the current mainstay for vesicle separation/collection, UC still has its downfalls [15]. Of increasing concern within the field is: (1) the inappropriate application and interpretation of UC in the analysis of EVs; (2) the rotor-induced variability due to different k factors; (3) the failure of investigators in reporting the k factor and rotors used in their experiments; and (4) the replacement of UC by new and unverified techniques.

UC tends to be the most commonly practiced technique for EV isolation. Most UC protocols consist of a series of centrifugation steps to remove cells and debris, (300–3000× *g* 5–30 min), separate small and large EV populations (10,000–20,000× *g* for 30 min or filtration using a 0.2–0.8 um filter) and pellet EVs (100,000–167,000× *g* for 1–18 h) [38,39]. However, a major limitation of UC is the co-pelleting of cell-free DNA (cfDNA), proteins, ribonucleoproteins (RNP), or lipoproteins (LPP) with the sedimented EV sample causing contamination [38]. In addition, concerning EV heterogeneity, UC isolation methodology captures all EVs from a sample and is unable to differentiate between EVs derived from specific cell types [38,39].

Density gradient centrifugation, including sucrose, or the more recently validated iodixonal (OptiPrep) gradients, have shown to reduce co-pelleting consequences of standard UC. This is especially advantageous to purifying samples with high levels of protein aggregates, such as human biofluids. Size exclusion chromatography has also proven successful in fractionating EV populations by size with the major drawback of loss of sample with each successive purification step [38]. Again, though these methods allow for better purification than UC, all vesicles are separated on size alone, regardless of cell origin.

As interest in the EV field grows, many commercial EV and exRNA isolation kits have become available that exploit variations in polyethylene glycol or sodium chloride precipitation strategies (Exoquick, SBI, Life Technologies, Norgen Biotek, Exiqon). Similar to UC, drawbacks of precipitation technologies are co-precipitation of undesired macromolecules circulating in biofluids. PP is also not suitable for large starting material [38].

Immunoaffinity purification utilizing antibody-coated beads or heparin-coated agarose (ExoCap, Microfluids, uNMR) has proven to efficiently isolate subpopulations of EVs from cell culture media and unprocessed biofluids. While affinity-based methods are the most beneficial to extract homogenous EV profiles, this method is confined to isolating only one subset of EVs expressing a specific antigen. Since the diversity of EVs remains to be investigated, it is uncertain how representative a subset of EVs isolated using one antibody, or even multiple, is to the entire EV population [38,39].

## 4. Characterization of Heterogeneous Populations of EVs

In order to address the heterogeneity issue within the EV field, it is paramount for investigators to analyze EV subpopulations’ biological functions independently. This can be accomplished, for example, through the use of immune-affinity bead pulldown assays. To accelerate biomarker discovery and elucidate the roles of EV in tissue maintenance, future studies must aim to improve cargo characterization [40]. It is paramount to take advantage of the rapidly growing number of methods in proteomics and genomics in order to fully determine the RNA and protein content of vesicles. Combining results from multiple omics will allow for a more comprehensive understanding of EVs. 

It is also crucial to learn whether all EVs taking part in intercellular communication contain RNAs/DNAs or whether nucleic acids are transported by specific EV subtypes. Finally, researchers must determine whether subtypes classified based on their respective functions exist. Experiments focusing on single EV analysis and employing techniques allowing for the enrichment of distinct EV subpopulations will help us to better understand EV mediated intercellular signaling.

## 5. Single EV Analysis

Single EV analysis is highly challenging due to their nanometer sizes. So far, optical and non-optical approaches are mainly used to characterize single EV. Here, we review various mainstream technologies (Table 1).

## 6. Optical Methods

### 6.1. Optical Microscopy

Optical microscopy has greatly facilitated our understanding of cellular biology [78]. Due to the optical diffraction barrier, it is challenging to accurately locate and image EVs (<100 nm) by using conventional optical microscopy with a resolution of several hundred nanometers [78]. Recently, a series of novel super-resolution imaging techniques are developed to overcome the diffraction limit, such as near-field scanning optical microscopy (NSOM) [79,80], stimulated emission depletion (STED) microscopy [81,82], and single molecule localization microscopy (SMLM) [83]. The SMLM technique is able to control the fluorescent molecules between an ON and OFF state, thus to isolate and localize individual fluorescent molecules, e.g., photoactivated localization microscopy (PALM) [84], and stochastic optical reconstruction microscopy (STORM) [85,86]. Nowadays, these super-resolution microscopies can provide up to an order of magnitude improvement in spatial resolution over conventional fluorescence microscopy and have been applied for qualitative or quantitative analysis of subcellular structures [41,87]. Chen et al. used immunofluorescence probes to label CD63 and HER2 of exosomes secreted from HeLa and SKBR3 cells [42]. Then utilizing PALM/STORM imaging technique (20–50 nm resolution) with these high density dual-color photoswitchable dyes (∼2 nm in size) to simultaneously track human breast cancer-derived EVs, lysosomes, and membrane receptor proteins on EV membranes. Such single-molecule colocalization super-resolution imaging techniques can pave the way to observe the interaction between cancer-derived EVs and normal cells. It also has a great potential in the investigation of mechanism of EV-mediated cancer metastasis. Daaboul et al. adopted interferometric reflectance imaging technique to multiplex phenotype single EVs captured on a microarray chip. This digital detection can analyze EVs directly from human cerebrospinal fluid [43]. Cell-derived EVs, EV movements, and miRNAs inside EVs are observed by SMLM technique. Moreover, dual-color SMLM based dynamic tracking revealed that EV cargos can protect miRNAs inside to prevent enzyme degradation during transfer and then release them into recipient cells [44]. New fluorophores, such as silicon quantum dots (Si QDs), possess a fluorescence blinking behavior, making them an excellent candidate for SMLM and indeed realizing super resolved optical imaging of EVs [45]. Adopting the SMLM technique coupled with custom nanoparticles can provide new insights in tracking the EV motion and encapsulated molecules of EVs involved in the pathway of intercellular communication.

### 6.2. Flow Cytometry

Flow cytometry and the fluorescence-activated cell sorter (FACS) are routine tools used in biomedical research and clinical diagnostics [88,89,90]. In scattering flow cytometry, polystyrene or latex microbeads with specialized sizes and concentrations are required for the quantitation and profiling of heterogeneous EVs. Therefore, to analyze EVs relying on the scattering flow cytometry alone is inefficient. In addition, its detection limit is usually ≧300 nm [46]. Fluorescence flow cytometry is a laser-interrogated particle fluorescence method, more sensitive than scattering flow cytometry because the emitted fluorescence intensity is higher than light scattering for particle size less than 300 nm [74,91]. It greatly facilitates the cell study in both physical (e.g., size, shape) and biochemical properties (e.g., cell cycle analysis, DNA contents, intracellular cytokine measurement) with a nondestructive and quantitative manner. However, its straightforward applicability for EV studies is hampered by the small size, polydispersity, and low refractive index of vesicles [92]. In general, the forward scattering signal can provide roughly information of particle sizes, whereas the side scattering signal can provide information about smaller particles or granularity of internal structures [93,94]. Some studies used beads-free flow cytometry through labeling lipophilic dye and antibodies on EVs for high-end cytometer analysis [47,95,96]. However, this kind of strategy still has restrictions, mainly due to the limited sensitivity and resolution of flow cytometers. The imaging flow cytometry equipped with an extra 60× objective and CCD can advance the detection sensitivity on EV analysis since this novel method facilitates visual confirmation of fluorescent events [48,97]. Adopting immuno-magnetic or latex beads (μm size) to isolate and concentrate EVs from samples is a much simpler strategy for subsequent bulk EV flow analysis [92,98].

### 6.3. Dynamic Light Scattering (DLS)

DLS is a technique that reflects scattering light intensity distribution under Brownian motion of suspended particles within a time period [50]. It can provide information of mean particle size and polydispersity index (PDI) [51]. It has been used to characterize the size of EVs derived from cells [52,99,100]. The diameter of the EVs is obtained from application of the Stokes–Einstein equation. However, in polydispersed suspensions, larger particles (e.g., microvesicles) generate more scattering light than smaller EVs, resulting in the inaccuracy for EV analysis and a bias toward the detection of larger particles. Additionally, it is unable to provide any biochemical information about cellular origin of EVs.

### 6.4. Nanoparticle Tracking Analysis (NTA)

NTA relies on using dark-field microscope together with blue laser excitation and equipped CCD/CMOS to direct observe and count the light scattering events of particles within a period of time [53,54]. Through tracking the Brownian motion of suspended single vesicles, the NTA software collects data on multiple particles and calculates the hydrodynamic diameter of each vesicle using Stokes–Einstein equation. Conventional NTA can deliver much more accurate information of both vesicle size distribution and vesicle concentration in solution [52,53,101,102]. Fluorescent NTA (F-NTA) is the other advanced method to analyze EVs presenting in a heterogenous sample [103]. This method specifically and efficiently labels intact EV surface with a proprietary dye or quantum dots (QDs) [104], resulting in exclusion of membrane fragments, protein aggregates, and background particles without probes. This strategy can increase the entire NTA signal-to-noise ratio because the fluorescence intensity is considerably higher than the light scattering intensity. It gives more accurate EV NTA data indicating the EV-specific populations in sample rather than all particles as typically provided by conventional non-fluorescent NTA. The resolution of fluorescent NTA in phenotype EVs can be down to ∼50 nm, thereby improving current characterization techniques [54].

### 6.5. Raman Spectroscopy

Raman spectroscopy is a powerful optical technique for providing the molecular vibrational state of a molecule structure with minimal sample preparation time. This inelastic scattering process is complementary to the infrared absorption spectroscopy [105]. Raman bands arise from a change in molecular polarizability when samples are irradiated by laser. The detected scattering spectrum can represent a unique optical fingerprint of a molecule. Therefore, it can be applied in non-invasive characterization and identification of molecules existing in a biological system. In the conventional confocal micro-Raman spectroscopy, the spatial resolution is ~1μm [106]. Generally, the optical intensity in Raman scattering is very low and difficult to distinguish. Thus, in experimental feasibility, various metal nanostructures are used to enhance the local electric field to amplify the weak Raman signals for better analysis, known as surface-enhanced Raman spectroscopy (SERS) [55]. The label-free, nondestructive, and noninvasive characteristics of SERS enable its application to biosensing. Some studies used Raman spectra to analyze single or bulk EVs and reveals their subpopulations distributed among multiple cancerous and noncancerous cell lines with variability related to membrane content [56,57]. Other studies applied different nanoparticle SERS and component analysis methods to clearly distinguish vesicles’ origin [107,108,109]. In combination with multivariate statistical analysis, the sensitive SERS method can provide an optical noninvasive cancer diagnostic tool at the single EV level.

### 6.6. Stimulated Emission Depletion (STED) Microscopy

STED microscopy is a fluorescence microscopy technique built on the basis of confocal laser-scanning microscopy [110,111]. Being different from confocal microscopy that all fluorophores are excited within the laser focal spot of sample, STED microscopy uses a second laser to generate energy-matching photons to switch off excited fluorophores in the outer regions of diffraction limited excitation focus [112]. It can provide a spatial resolution down to 16 nm in the focal plane, corresponding to about 1/50 of the employed laser wavelength [113]. Its subdiffraction resolution overcomes the diffraction limited resolution of confocal microscopy, but still possesses the merits of conventional fluorescence microscopy, including optical sectioning of a sample at different focal planes and molecular recognition specificity with a high sensitivity. The STED super-resolution imaging technique has been used to profile the expression of tagged protein receptors at the surface of EVs. Grapp et al. utilized two-color STED technique to assess the EV size and confirm the vesicle-specific colocalization of folate receptor (FR)-α with EV marker Alix [58]. Willig et al. applied STED microscopy to resolve individual vesicles in the synapse and observed that the vesicular resident protein, synaptotagmin I, congregated in isolated patches on the presynaptic membrane, regardless of whether nerve terminals were strongly or gently activated. This super-resolution imaging can dramatically help us to understand the EV morphology and protein distribution condition in vesicle recycling [59].

### 6.7. Fluorescence Correlation Spectroscopy (FCS)

The FCS is a confocal microscope based single molecular analytical technique that provides temporally quantitative localized measurements of fluorescence intensity fluctuations of labeled molecules in a femtoliter observation volume [60]. It offers information of diffusion coefficient, size, shape, and concentration of detected particles and/or molecules. Its detection limit can be down to 50 nm that enables one to decipher biomolecular interactions [61]. FCS has been used to determine the concentration and mobility of protein receptor diffusion of vesicles on plasma membrane of cells [114]. Combining with highly efficient purification procedures, single fluctuation analysis with a single-molecule sensitivity enables to provide in-depth characterization of size and protein expression level on single vesicles [62,63].

## 7. Non-Optical Methods

### 7.1. Transmission Electron Microscopy (TEM)

With negative staining, cryo-TEM is a widely used tool for EV imaging in determination of its morphology, size, and structure [64,65]. Typical morphologies of EVs is a cup-shape. The function-dependent morphology and structure determination of EVs in necessary important in medical and pharmaceutical fields. Through negative staining and immuno-gold labeling of a specific protein on EVs, we can clearly verify the morphological distribution of target protein and structure of EV by TEM techniques [66,67]. Some studies used cryo-TEM to characterize individual EVs in their native state in fresh human plasma [64,115]. Their study indicates that only a small fraction could be identified as EVs, however, most particles are lipoproteins.

### 7.2. Atomic Force Microscopy (AFM)

AFM is often referred to as scanning probe microscopy (SPM). This topography imaging technique has a very high subnanometer resolution, more than 1000 times better than the optical diffraction limit [68,69,70]. It maps the interaction force, e.g., van der Waals, electrical, magnetic, or thermal, between the sample and sharp tip (~50 nm), also provides localized properties, e.g., height, friction, or magnetism. Raster scanning over a small area of sample is required in image construction. To image biological samples (DNA, proteins, fibrils, vesicles), an immobilized sample on a very flat surface with roughness less than 0.5 nm is necessary. Its ability to monitor biological samples in aqueous fluids offers the merit of preserving sample properties in their physiological state [71,72]. The most adopted immobilization strategies for EV visualization are bare surface of mica/glass substrate, poly-l-lysine/APTES functionalized mica, or specific antibody-coated mica/silicon surfaces [116]. AFM has been applied to directly detect platelet-derived vesicles from blood with elevated EV accounts due to cardiovascular disorders and cancer, then analyze their morphology, mechanical properties, and size distribution [68,73]. It can be an alternative novel method for the sensitive detection of defined subsets of vesicles in nanosize range, far below the lower detection limit of conventional flow cytometry. The AFM technique enables to offer the particular ID information of size distribution, morphology, mechanical properties, biomolecular load of EVs derived from specific subpopulations of cells. These characterizations can be categorized and used as an effective tool to discriminate EVs secreted from healthy and tumor cells, ultimately with the aim of identifying plasma-derived EVs of unknown origins.

### 7.3. Impedance-Based Flow Cytometry (IFC)

IFC based on the Coulter principle provides a sensitive approach that allows fast and automatic single-particle analysis revealing the physical properties about particles size distribution, concentration, and surface charge [74,75]. The EV membrane is semi-permeable bilayer of lipids and proteins encapsulating the cellular content. An intact membrane is not highly conductive and acts electrically as a combination of capacitor and resistor. Typically, vesicles in electrolyte solution flow through a narrow sensing aperture, where each vesicle displaces its own volume of electrolyte. The displaced volume increases the electrical resistance across the circuit, generating a voltage pulse where the height of each pulse is proportional to the volume of the particle. In comparison to conventional flow cytometry, IFC is independent of refractive index of measured vesicles. However, it is able to resolve only EVs that are larger than 300 nm, e.g., blood-borne vesicles [76,77]. Currently, smaller instruments are available with the involvement of microfluidics and lab-on-a-chip technology [117]. The detection limit for vesicles can be down to 70 nm when using a microfluidic IFC [91,118]. The IFC methodology does not offer information on morphology, biochemical composition, or cellular origin of vesicles, but it has flexibility to be further combined with light scattering and fluorescence flow cytometry.

### 7.4. Examples of Single Cell RNA Techniques That Can Be Applied to EV Analysis

EVs have been discovered to contain RNA, allowing the intercellular transfer of genetically encoded messages [119]. Amid the growing interest in EV RNA, current understanding of the underlying mechanisms that drive and regulate RNA uptake by EVs is limited, and there exist various technical challenges within EV RNA analytics [12]. Analysis methods, PCR and RT-PCR for single cell RNA analysis, can directly be applied in EV RNA analysis [120]. Deep sequencing-based expression analysis using microarray technology has been applied to study the EV RNA [121]. However, it is not possible to identify new sequences if only adopting known sequences as targets in microarray. Additionally, there is a risk for probe cross-hybridization when using microarrays. Droplet microfluidics can also be used to analyze RNA. Similarly to the PCR case, RT-PCR amplification in microfluidic droplets is initially demonstrated on chip with immobilized droplets on a thermal plate and a fluorescent readout to detect droplets initially containing an RNA target molecules [122]. Recently, quantitative PCR and digital PCR have been applied to quantitative analysis of EV RNA [123,124]. Moreover, the microfluidic chip is employed to analyzed mRNA levels of enriched tumor EVs obtained from patient blood [125,126]. If these methodologies validated on a larger cohort of patients, they may be used to predict cancer treatment drug response in patients.

## 8. Examples of Single EV Experiments

Recent studies have employed single EV analysis methods to characterize the cargo of individual vesicles. In an experiment by Smith et al., Raman spectroscopy was used to investigate the chemical composition of single exosomes from eight different cell lines [56]. Both cancerous and non-cancerous cell lines were analyzed. The spectra of individual vesicles within each cell line varied significantly and were determined to cluster into four distinct categories that were consistent across all cell lines. Using principal components analysis (PCA), the group found that each cluster differed from the rest based on membrane composition. Specifically, cholesterol content, expression of surface proteins, and relative expression of phospholipids to cholesterol were hypothesized to be the main sources of variation. These results suggest that several classes of exosomes exist that vary in chemical composition and biological purpose. Exosome class is most likely influenced by factors such as the role of the cell from which it forms and its microenvironment. Higher quantities of exosomes were isolated from cancerous cells as compared to normal cell lines, suggesting a more profound function for these particles in cancerous environments.

Carney et al. report the application of multispectral optical tweezers (MS-OTs) to single EVs for biochemical fingerprinting of vesicle subpopulations [127]. MS-OTs is a technique that combines laser trapping Raman spectroscopy (LTRS) with a fluorescence imaging system and has been previously described in the characterization of individual whole cells [128]. In this study, it was utilized to compare the chemical composition of single exosomes isolated from in vitro mesenchymal stromal cells (MSCs) to those captured from in vivo plasma. The study revealed a distinct molecular fingerprint for CD9-positive EVs found in MSCs as well as plasma. This CD9^+^ subset consists of vesicles that bind fluorescently labeled antibodies against CD9, a membrane protein of the tetraspanin family often used as an exosome biomarker [129]. Carney et al. found that this subpopulation of EVs display less chemical heterogeneity as well as reduced component concentration in comparison to the bulk population averages characterized by classic ultracentrifugation techniques.

Sharma et al. describe the first application of high-resolution atomic force microscopy (AFM) for the quantification and characterization of single vesicles. Their comparison of exosomes found in the saliva of oral cancer patients to those isolated from healthy controls revealed that cancer extracellular vesicles are significantly increased in size, display irregular morphology, and a greater level of inter-vesicular aggregation [130]. The study also found that exosomes from oral cancer patients demonstrated significantly increased CD63 surface densities. These findings present AFM as an effective approach for quantifying and identifying the structure and surface protein constitution of exosomes. This technique has the potential to be used as a method for tracing cancer progression through periodic exosome measurements and can be applied to the study of other cancers as well.

Exosome research has grown tremendously in recent years after discoveries that these particles transport functional mRNA, miRNA [131], and DNA [132] between cells, and are packaged for highly specific intercellular communication, including commutation as a mode of disease pathogenesis [133,134]. While traditional studies tend to rely on bulk analyses of EVs [56], many more recent experiments focus on fractionating and studying subpopulations of EVs, while some aim at studying single exosomes or larger microvesicles. Fractionation and single EV analysis studies currently rely on a wide array of varying physical separation techniques and technologies.

A recent study by Haiying Zhang et al. sought to analyze EV subpopulations after separation by asymmetric flow field-flow fractionation (AF4), and to establish and optimize AF4 parameters and protocols for small EV (sEV) fractions isolated from numerous cancer and normal cells [135]. The study showed that asymmetric flow field-flow fractionation (AF4) can be used as an analytic tool for isolating EVs and addressing nanoparticle heterogeneity. Two exosome subpopulations and one new nanoparticle population were identified using this technology: large exosomes (“Exo-L”, 90–120 nm), small exosomes (“Exo-S”, 60–80 nm), and ‘exomeres’ (~35 nm), respectively. Each subpopulation had distinct *N*-glycosylation, protein, lipid, DNA, and RNA profiles, while cargo from these subsets seemed to reflect diverse organ biodistribution patterns. Interestingly, Exo-L exhibited an enrichment of proteins associated with the mitotic spindle and IL-2/STATS signaling pathways, while Exo-S where characterized by enrichment of proteins associated with endosomal functions and secretion pathways. On the other hand, small exomeres showed enrichment of metabolic enzymes and proteins associated with hypoxia, microtubules, coagulation, glycolysis, and mTOR signaling. Comparatively, Exo S/L were enriched in membrane-associated proteins, which were depleted in exomeres, while ESCRT and SNARE related proteins found in Exo S/L.

In a 2015 study, Zachary J. Smith et al. studied the chemical composition of single EVs using Raman spectroscopy, analyzing vibrational, rotational, and other low-frequency molecular characteristics of the particles [56]. While single exosomes isolated from the same cell type exhibited high spectral variability, single exosomes could be clustered into four distinct groups that were not cell-line specific, differences between these groups primarily due to differing membrane compositions. Populations varied largely in cholesterol content, relative expression levels of phospholipids to cholesterol, and surface proteins. Exosome subpopulations seemed to be shared among cell types, suggesting distributed exosome functionality. Additionally, these differences may be attributed to the specific roles of EV subpopulations in both normal cell functionality and in carcinogenesis, and may provide diagnostic potential at the single exosome level using Raman spectroscopy or other methods.

In addition to efforts at implementing known methods to study subpopulations and single EVs, novel devices are also being engineered to study these particles with greater precision. For instance, Kyungheon Lee et al. made recent progress in multiplexed profiling of single EVs using an original microfluidic device developed for single EV analysis [136]. The method involved biotinylating EVs before capturing them on the device’s neutravadin-coated glass surface. Use of the chip enabled greater control of experimental conditions, such as flow rate and incubation time, to facilitate downstream washing and staining steps while minimizing the risk of sample loss. Once made stationary in the device, EVs could be more efficiently imaged analyzed, as the vesicles were stained using fluorescent antibodies recognizing ubiquitous EV markers or tumor markers.

Previous research states that conventional flow cytometry (FACs) cannot efficiently differentiate biological nanoparticle samples from background due to their small size, quantity of surface antigens, and a lack of sensitivity. Lof et al., proposed a protocol to quantify and characterize EVs by FACs in conjunction with an in situ proximity ligation assay (in situ PLA or ExoPLA) to optimize fluorescent signatures [137]. The ExoPLA uses multicolor staining of EV surface proteins to amplify fluorescence signals so EV samples can be detected above standard FACs background. EVs were collected using a bead-based pull down (via CD63 capturing antibody) so as to immobilize vesicles for staining before amplification of four PLA probes and analysis by FACs. PLA probes used commonly targeted vesicle surface markers such as CD26, CD10, CD13, and Cathepsin B. BD LSRFortessa and the BD LSRII flow cytometer were used to quantify and characterize EV samples. Despite challenges with nanoparticle analysis and technological limitations of conventional FACs, the reality that EVs play a crucial role in disease progression and diagnosis highlights the urgency for sensitive and nanospecific methods of analysis. In situ PLA with FACs provides two advantages for EV analysis: specific EV surface protein targeting, and sufficient fluorescent amplification of samples for detection. 

Invading GBM cells and cancerous biological material move from cancerous cells through the parenchyma interacting and changing the neuronal landscape, communication of cancerous biological cargo is another avenue of EV exploration. Wei et al. developed a fractionation and sequencing protocol to optimize quantitative analysis of extracellular RNA (exRNA) associated with EVs and ribonucleoproteins (RNPs) [138]. Wei used four patient derived GBM stem-like cell (GSC) populations for heterogeneous EV and RNP exRNA profiling. Multifiltration and low impact centrifugation isolated desired RNPs and EVs of interest, high quality RNA profiles were analyzed by Aligent 2100 Bioanalyzer, and RNAseq was run for exRNA library construction. Wei’s protocol provides a low impact, accurate separation of RNPs and EVs, with higher RNA yield, and uses scalability to create size-similar populations within a sample. Limitations are that this protocol is time consuming and separation is based solely on size which makes removing other desired EVs from the filter membrane difficult and could potentially alter sample structure. Wei et al. was able to create an exRNA library despite having no adequate quantitative assessment for extracellular RNAs. Their findings and accumulated data allow for predictions of tumor associated GBM exRNAs. This data provides a significant insight into the genetic heterogeneity of GSC associated exRNAs, and the way in which tumor cells utilize a variety of mechanisms to sort and export exRNA by EVs and RNPs into the surrounding neuronal environment.

## 9. Tumor EV Heterogeneity

EVs have been shown to be released by many different types of cells of both hematopoietic and non-hematopoeitic origin [139,140]. The proposal of EV secretion in vivo came from observations that vesicles from prostate epithelial cells correspond in size to the intraluminal vesicles of storage vacuoles (the equivalent of MVBs) in these cells [16,141]. EVs with properties similar to those of exosomes have been successfully isolated from diverse biofluids, including semen [141,142,143], blood [115,144], urine [145], saliva [146], breast milk [147,148], amniotic fluid [149], ascites fluid [150], cerebrospinal fluid [151], and bile [152]. The isolated vesicles in these studies were thought to represent exosomes because of the composition of their protein contents. However, circulating EVs are likely composed of both exosomes and microvesicles (MVs); in fact, the suggestion that single cell types release both exosomes and MVs has been demonstrated in platelets, endothelial cells, and breast cancer cells [16]. Thus, we must focus on establishing methods that will allow for researchers to clearly distinguish between exosomes and MVs. Currently, comparisons made based on properties such as size, morphology, buoyant density, and protein composition seem insufficient for proper classification [153]. It is possible that the origins of EVs might be better understood by looking at the interference of the molecular mechanisms required for EV formation and cargo sequestration—modern developments that will allow for new avenues to resolve their respective functions [16]. Clear discrimination between vesicle types, in addition to the availability of well-established technologies for purification of certain EV subtypes, are lacking. In a circular fashion, it is typical for many to connect the issue of vesicle nomenclature to the process of vesicle collection itself [15,16]. Of the various techniques which have emerged in the past decade, differential centrifugation remains the ‘gold-standard’ for the isolation and collection of EVs. In fact, many researchers use this technique to define and distinguish the microvesicle (pellet at ~10,000× *g*) and exosome (pellet at ~70,000–100,000× *g*) as separate secreted EV subtypes. However, conventionally, the end result remains a very heterogeneous pellet of different vesicle types enriched in parallel [14]. As a result, members of the International Society of Extracellular Vesicles (ISEV) recommend discriminating vesicles under study in experimental enriched fractions as EVs, rather than as exosomes, MVs or apoptotic bodies [14,26]. 

Previous research has mainly focused on comparative analysis of classic EV subtypes (i.e., exosomes, MVs, and apoptotic bodies) [154,155,156,157] and of EVs released from the apical and basolateral surfaces of organoids, such as those derived from colon carcinoma cells [158]. However, some studies reveal distinct molecular compositions and biological properties within EV subpopulations [3,159], further complicating our understanding of EV mediated intercellular signaling.

Almost all cells release different types of EVs. In cancer patients, tumor-derived EVs have been found in diverse body fluids, such as blood and cerebrospinal fluid [160]. It is increasingly evident that EVs have the ability to transfer molecular information, which explains their key role in regulating cell–cell communication [161]. In their early landmark paper, Valadi et al. showed that EVs released by mast cells contain messenger (m)RNA molecules that can be translated into functional proteins upon transport to recipient cells [131]. As previously described, EVs are now known to shuttle many other functional biomolecules, including proteins, lipids, miRNA, long non-coding RNA and DNA [162], between different cell types present in multicellular organisms. Transferring of vesicular content, as well as surface-bound receptors and their ligands, may influence the phenotypic behavior or fate of recipient cells (e.g., by inducing differentiation or de-differentiation, or by promoting apoptosis) [161]. It is unclear how long EV-mediated effects last; however, some postulate that their duration may be dependent on biomolecule type being transferred. Moreover, it is possible that EV-mediated transfer of DNA, miRNA, mRNA, and/or transcripts leads to epigenetic reprogramming of target cells—ultimately resulting in a stable, long-lasting behavioral change [161]. On the other hand, vesicular transfer of membrane receptors (e.g., proteins that are considered to actively turnover) may only alter the phenotype of recipient cells temporarily [161].

In the context of tumor-derived EVs, vesicles are comprised of proteins, lipids, and genetic material contributing to cancer progression. Furthermore, cellular uptake of tumor-derived EVs can change a noncancerous cell’s otherwise normal phenotype to a cancerous state. Studies have reported that oncogenes are not only incorporated into EV-cargo, but also act to upregulate tumor cell vesiculation [163]. Therefore, heterogeneous populations of EVs can have potential implications in EV-mediated local and systemic transmission of phenotypic behavior, such as the malignant transformation of normal cells.

A large contributing factor to the challenges of EV heterogeneity characterization is a lack in validated isolation platforms that support EV subpopulation enrichment. A thorough comparison of current EV isolation methods and commercial kits demonstrates significant differences among the available protocols, dependent on type of biofluid, sample volume, and fraction of exRNA of interest [38]. Currently, there is no universal isolation technique suitable for all studies. Broadly speaking, among the most widely reported literature for EV and exRNA isolation, proprietary platforms can be classified into four categories: ultracentrifugation (UC); precipitation using chemical polymers (PP); fractionation, including density gradient centrifugation (DG) or size exclusion chromatography; and immunoaffinity purification [38].

## 10. Concluding Remarks

Amidst the recent explosive rise in EV research, there still exists significant challenges in terms of EV population heterogeneity—mostly due to the lack of technologies that allow for single EV analysis. Throughout this review, we have provided an overview of recent developments in vesicular research and its respective technologies that, with further improvements, can help answer some of the pressing questions that still remain. Future work and advanced platforms that can study EVs at the single level are a major need and will hopefully become available in the near future. 

## Figures and Tables

**Table 1:** Overview of methods for single EV analysis.

Methods	Strength	Limitation	Reference
Optical microscopy	High resolution (20–50 nm) imaging provides single molecule information on EVs, records EV movements and local interactions with cells.	Target proteins on EVs may be disrupted in labeling. Fluorophore induced dimerization or photobleaching may happen.	[41,42,43,44,45]
Flow cytometry	Enable fast, multiple, and high throughput detection of bulk EVs and single EV. Applicable to clinical research. Providing extra value in imaging measured EVs.	Possible high background signal in analyzing fluorophore labeled EVs due to the size is less than 200nm and the heterogeneity of refractive index of EVs. The bead calibration with known sizes and counts is required to permit quantitation and delineation of heterogeneous EVs.	[46,47,48,49]
Dynamic light scattering (DLS)	A fast and non-invasive approach in analyzing EVs.	Unable to provide any biochemical information about cellular origin of EVs. Possible inaccurate EV analysis due to various sizes of EVs. Stable temperature and solvent viscosity are required for obtaining reliable results.	[50,51,52]
Nanoparticle tracking analysis (NTA)	A fast and easy approach for counting bulk EVs. No shrinkage artifacts due to fixation.	Low dynamic range in differentiating EV sizes.Low sensitivity to fluorescent signals.	[49,52,53,54]
Raman spectroscopy	A label-free, non-destructive, and non-invasive method for single or bulk EV analysis. Unique molecular information can be obtained.	High background and weak intensity signal limits the dynamic range of measurements. Fabricated substrates and nanoparticles for signal enhancement are required.	[55,56,57]
Stimulated emission depletion (STED) microscopy	A high-resolution imaging technique in assessing EV sizes and localized proteins of single EV.	High quality sample preparations and protein labeling with fluorophores are required. Not straightforward for fast and high throughput EV analysis.	[58,59]
Fluorescence correlation spectroscopy (FCS)	Single molecule measurements with high spatial and temporal resolution, short analysis time, and little sample consumption.	The diffusing fluorescent particles must be able to move between the high and low excitation intensity regions. The volume of the laser-excited observation region must be smaller than the volume of confined particles.	[60,61,62,63]
Transmission electron microscopy (TEM)	High resolution imaging in determination of morphology, size, and structure of EVs.	Hard to be applied for high throughput molecular profiling of EVs. High quality and pure EV preparation is required. Unable to provide information of EV from different origin.	[64,65,66,67]
Atomic force microscopy (AFM)	A very high-resolution imaging technique. Able to provide size, distribution, morphology, mechanical properties, biomolecular load of EVs derived from specific subpopulations of cells in their physiological state.	Slow speed in measurements and limited imaging area. Unable to provide the molecular information inside EVs. Results are influenced by AFM probes.	[68,69,70,71,72,73]
Impedance-based flow cytometry (IFC)	A fast and sensitive approach in providing particle size distribution, concentration, and surface charge.	Unable to offer information on morphology, biochemical composition, and cellular origin of EVs. The dynamic range of size measurements relies on the aperture diameter of flow chamber.	[74,75,76,77]
